# EVALUATING THE WYOMING GWEP PARTNERSHIP

**DOI:** 10.1093/geroni/igac059.886

**Published:** 2022-12-20

**Authors:** Catherine Carrico, Irene Lujan, Trena Anastasia, Christine McKibbin

**Affiliations:** University of Wyoming, Laramie, Wyoming, United States; Eastern Shoshone Tribal Health, Ft. Washakie, Wyoming, United States; Thrive Alive, Masonville, Wyoming, United States; University of Wyoming, Laramie, Wyoming, United States

## Abstract

The goal of the Wyoming Geriatrics Workforce Enhancement Program (WyGWEP) is to utilize partnerships to improve health outcomes for older adults by developing a healthcare workforce trained to address unmet health and social determinant needs of Wyoming’s older residents. The purpose of this presentation is to report results from the novel mixed-methods evaluation of the WyGWEP partnership. Data were gathered from WyGWEP stakeholders through the Partnership’s Self-Assessment Tool (n=17; Center for the Advancement of Collaborative Strategies in Health, 2002), which assesses domains of Synergy, Leadership, Resource Sharing — Non-financial and Financial, Benefits and Drawbacks, Decision Making, and Administration/Management and Satisfaction. Semi-structured interviews (n=12) with partnership participants provided additional information and context. Evaluation results indicate that WyGWEP partners are highly satisfied across all domains of the Partnership’s Self-Assessment Tool. WyGWEP partners from Eastern Shoshone Tribal Health will share what has contributed to a successful collaborative. Additional results related to how this type of partnership has influenced participants’ ability to complete objectives and activities will be reviewed.

